# Galectin-3 Mediates TGF-β-Controlled Migration in Müller Glial Cells

**DOI:** 10.3390/ijms27188430

**Published:** 2026-09-21

**Authors:** Joshua Luis, Karen Eastlake, G. Astrid Limb, Peng T. Khaw, Hari Jayaram

**Affiliations:** National Institute for Health and Care Research Biomedical Research Centre for Ophthalmology, University College London Institute of Ophthalmology & Moorfields Eye Hospital, London EC1V 9EL, UKp.khaw@ucl.ac.uk (P.T.K.);

**Keywords:** galectin, gliosis, migration, TGF-β

## Abstract

Galectin-3 (Gal-3) is a ß-galactoside sugar-binding protein increasingly recognised for its role in systemic inflammation, fibrosis, and cancer. Within the retina, Müller glial cells (MGCs) are a major producer of Gal-3. This study aimed to investigate the impact of Gal-3 on the migration of MGCs. CRISPR-Cas9 knockout of Gal-3 resulted in slower cellular migration in MGCs (*p* < 0.001). This was partially rescued by the addition of exogenous Gal-3 (*p* < 0.01). The addition of Gal-3 inhibitor TD139 (*p* < 0.01) resulted in decreased cellular migration, whilst the addition of Neuraminidase 1 (*p* < 0.05) resulted in increased cellular migration. TGF-β treatment downregulated Gal-3 mRNA (*p* < 0.01) and protein (*p* < 0.001) expression; increased the mRNA expression of various sialylation enzymes; and resulted in significantly decreased cellular proliferation (*p* < 0.01) and migration (*p* < 0.001). These findings establish Gal-3 as a pro-migratory driver in MGCs under the regulation of TGF-β. Consequently, targeting the Gal-3/tissue sialylation axis represents a promising therapeutic avenue for mitigating pathological glial proliferation and migration in conditions such as proliferative vitreoretinopathy.

## 1. Introduction

Galectins (Gal) are a phylogenetically conserved family of lectins characterised by their high affinity to ß-galactosides. Whilst the majority of galectins form dimers, Gal-3 is structurally unique due to its capacity for oligomerisation [1]. This multivalent architecture is functionally critical, as it enables Gal-3 to cross-link multiple ligands and perform its roles, including the organisation of cell surface molecules such as β1-integrin and the formation of complex lattice structures [2,3]. The activities of Gal-3 extend significantly beyond its capacity to bind to ß-galactosides, as a range of additional distinct intra- and extra-cellular functions involving the central and N-terminal regions have been uncovered [4].

The functional efficacy of the C-terminal ß-galactoside binding region of Gal-3 is dependent on the glycosylation profile of its target ligands. Sialylation tends to inhibit Gal-3 binding in general; however, there is some evidence that Gal-3 can at times tolerate α2,3-sialyl linkages better than their α2,6-counterparts [5]. The attachment of sialic acid residues onto the terminal position of glycoconjugates is regulated by the sialyltransferases (SiaTs) family of enzymes. The specific roles of individual members of these SiaT families show significant redundancy and overlap and can also be organ-dependent [6]. Functional association between Gal-3 and tissue sialylation continues to be elucidated, as the coordination of this relationship may play a central role in cellular process such as proliferation and migration.

In recent years, the understanding of Gal-3 functions in systemic diseases has progressed rapidly, particularly in fields of oncology, fibrogenesis and inflammation [7,8]. From these studies, Gal-3 has emerged as a reliable biomarker in a range of disease states such as heart failure, liver fibrosis and various malignancies [9]. Beyond its role as a diagnostic and prognostic biomarker, studies have also investigated the mechanistic implications of increased Gal-3 concentration in these diseases. Whilst Gal-3 does not appear to be a molecule that is particularly disease-specific, itsupregulation consistently coincides with pro-inflammatory, pro-fibrotic, and pro-neoplastic states.

Within the eye, Gal-3 expression is observed throughout different tissues, where it has been shown to be integral in various processes of both normal physiology and disease, such as cell-signalling modulation, wound healing and immune cell activation [10]. The neuroretina is composed of intricately coupled tissues arranged in concentric neuronal layers; these are traversed radially by Müller glial cells (MGCs) across all layer to form individual neurovascular units [11]. Previous studies have demonstrated Gal-3 to be produced predominantly by astrocytes and MGCs within the neuroretina, with some contribution by microglia and circulating immune cells [12]. Immediately peripheral to the neuroretina, the retinal pigmented epithelium (RPE) has also been shown to produce Gal-3 at relatively high concentrations, where it performs important roles in maintaining RPE function [13].

As the sole cell type to span the entire thickness of the neuroretina, MGCs are in direct contact with all neuronal subtypes, the vasculature, and resident immune cells. This unique positioning allows MGCs to respond to changes within sub-compartments of the neuroretina and provide appropriate support to the high metabolic demands of its neighbouring neuronal cells [14]. Therefore, it is logical for MGCs to be a central regulator of a versatile molecule such as Gal-3 according to the needs of the micro-environment. Whilst Gal-3 upregulation is a known feature of retinal degeneration, where it has been implicated in apoptosis, inflammation and fibrosis [15], its exact functional contributions are likely to be highly nuanced and context-dependent. For instance, the presence of Gal-3 has been found to promote phagocytosis and reduce glial activation in retinal degeneration [16].

In response to injury cues, neuroretinal gliosis can lead to divergent functional outcomes, dictated by a complex array of underlying drivers [17,18]. The activities of cytokines such as TGF-β, TNFα and IL-1 have long been established to be involved in these diseases and likely play a central role in determining the course of gliosis [19]. Glial migration is a hallmark feature of this process, which allows movement of MGCs and astrocytes to the site of injury in their attempt to protect and repair the tissue [20]. Although there is accumulating evidence of Gal-3 involvement in the regulation of cell migration in extra-ocular tissues, comparable studies within retinal tissues are still lacking. Clinically, uncontrolled glial migration can be seen in diseases such as proliferative vitreoretinopathy (PVR) and proliferative diabetic retinopathy (PDR), where pathogenic fibrotic changes can extend into the vitreous and cause sight-threatening injury [21].

The aim of this study was to investigate the effects of Gal-3 on MGC migration, with a specific focus on the effect of cytokines such as TGF-β on the Gal-3/sialylation axis.

## 2. Results

### 2.1. TGF-β Decreases Proliferation and Migration of MIO-M1 Cells

Cytokines known to be active during retinal degeneration, including TNF-α, IL-1, IL-6, TGF-β and INF-γ, were added to the culturing media of MIO-M1 cells to investigate their effect on Müller glial cell proliferation and migration. All recombinant cytokines were added at 10 ng/mL, and the corresponding concentration of bovine serum albumin was added to the control samples. The 10 ng/mL concentrationwas selected based on previous proteomic analysis of proliferative vitreoretinopathy samples and subsequent optimisation [22]. The rates of cellular proliferation and migration were assessed using the MTT assay and the scratch assay, respectively.

**Figure 1:**
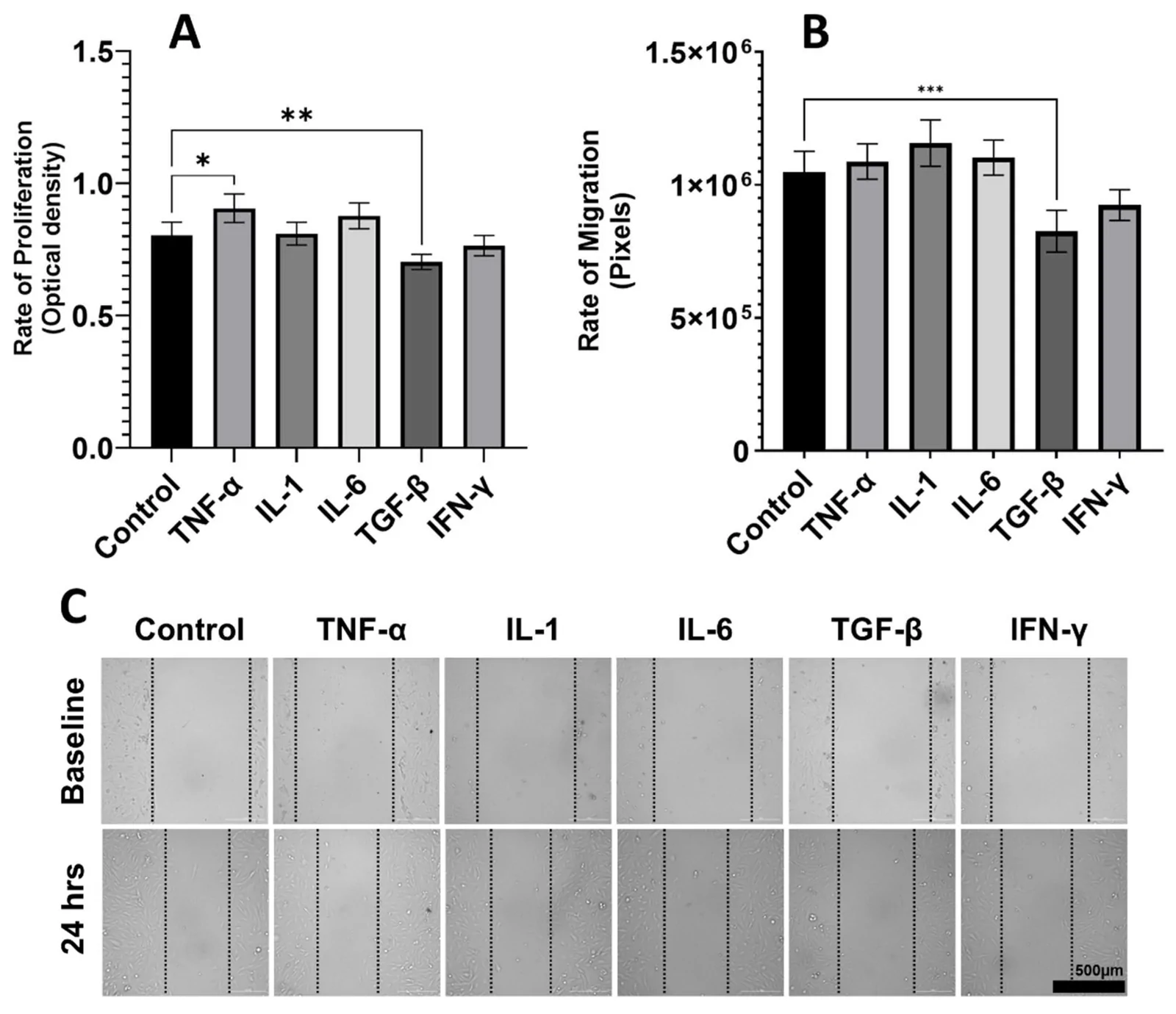
Effects of inflammation-associated cytokines on cellular proliferation and migration in MIO-M1 cells. (**A**) TNF-α increased the rate of proliferation (*p* < 0.05), whilst TGF-β decreased (*p* < 0.01) the rate of proliferation, *n* = 5. (**B**) TGF-β significantly decreased the rate of migration, *n* = 5. (**C**) Representative brightfield images of wells immediately and 24 h following scratch assay. *—*p* < 0.05, **—*p* < 0.01, ***—*p* < 0.001.

At 7 days following the addition of cytokines, TNF-α treatment resulted in a small but statistically significant increase in the rate of proliferation (OD 0.80 vs. 0.91, *p* < 0.05), whilst TGF-β treatment resulted in a small but statistically significant decrease in the rate of proliferation (OD 0.80 vs. 0.70, *p* < 0.01, Figure 1A). At 24 h following the addition of cytokines, TGF-β was found to significantly decrease the rate of migration (1.04 × 10^6^ vs. 0.82 × 10^6^, *p* < 0.001). IL-1, IL-6 and IFN-γ did not demonstrate any statistically significant effects on the proliferation or migration rates of MIO-M1 cells (Figure 1B,C).

### 2.2. TGF-β Downregulates Gal-3 Expression in MIO-M1 Cells

**Figure 2:**
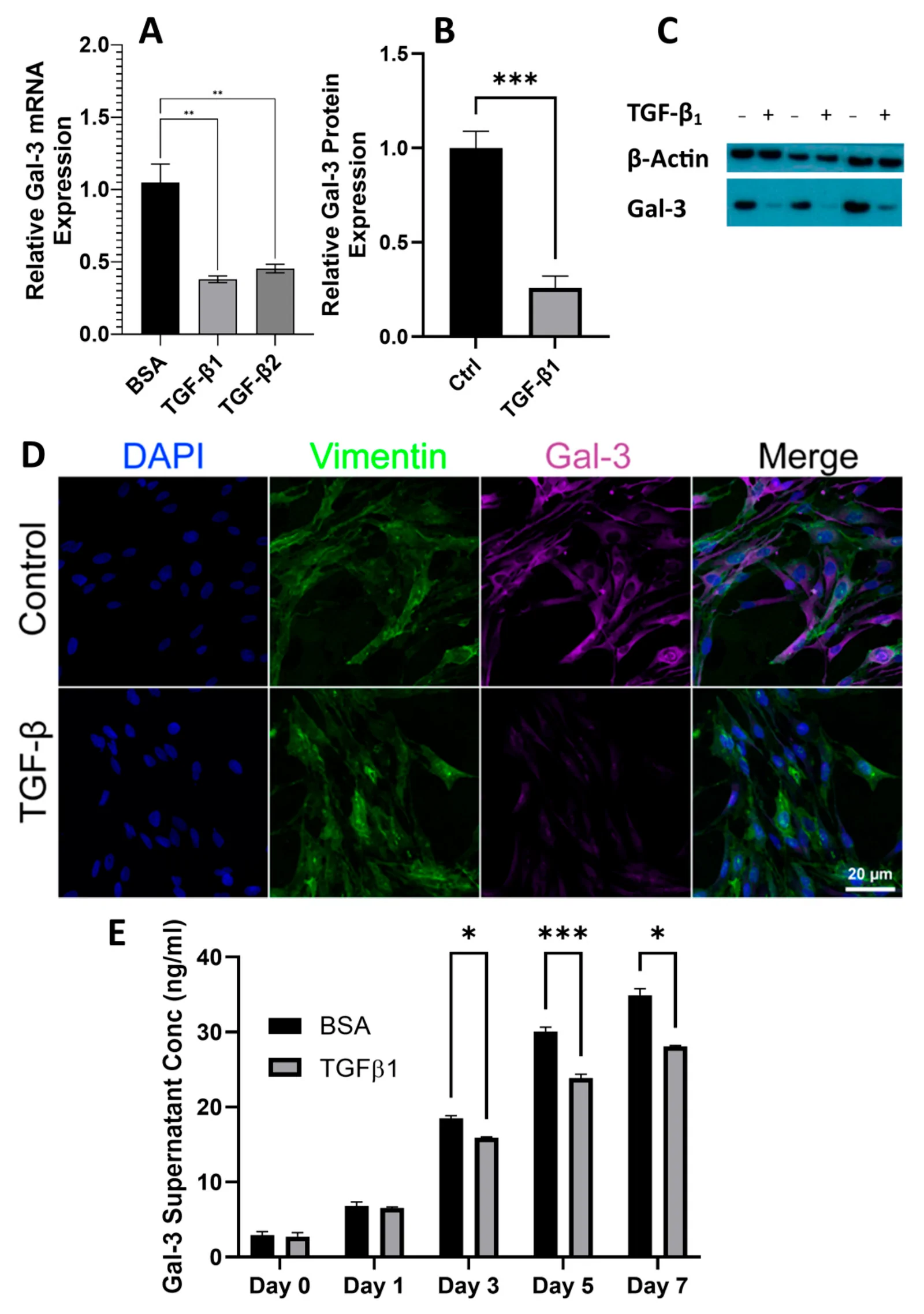
Treatment of MIO-M1 cells with TGF-β resulted in significant downregulation of Gal-3. (**A**) Gal-3 mRNA expression measured using qPCR showed significant reduction at 24 h for both TGF-β_1_ and -β_2_ isoforms. (**B**) Gal-3 protein concentration in cell lysates showed significant reduction following treatment with TGF-β_1_. (**C**) Representative Western blots. (**D**) Immunocytochemical staining of MIO-M1 cells showed reduction of Gal-3 protein expression within cell membranes following treatment with TGF-β as compared to control at 48 h. (**E**) Gal-3 protein concentration within the supernatant measured using ELISA showed significant reduction following treatment with TGF-β_1_ at 3, 5, and 7 days. *—*p* < 0.05, **—*p* < 0.01, ***—*p* < 0.001.

To determine whether TGF-β influenced the expression of Gal-3 by MGCs, 10 ng/mL recombinant TGF-β was added to MIO-M1 cells. At 24 h, a significant downregulation of Gal-3 mRNA expression was observed, which was confirmed for both the -β_1_ and -β_2_ isoforms of TGF-β (Figure 2A). Protein expression of cell lysates as measured by Western blot showed a similarly significant decrease at 48 h for TGF-β_1_ (Figure 2B,C). Immunocytochemical staining confirmed decreased Gal-3 expression as well, where Gal-3 was found to be mainly localised within the cell membrane of MIO-M1 cells, with reduced fluorescence seen within the nuclei (Figure 2D). Finally, the supernatant concentration of Gal-3 in cultured MIO-M1 cells, as measured by ELISA, increased steadily over 7 days, from 6.8 ng/mL on day 1 to 34.9 ng/mL on day 7 (Figure 2E). Following the addition of TGF-β_1_, no differences in Gal-3 supernatant concentration were seen at day 1, but significantly decreased concentrations of Gal-3 were found in the TGF-β_1_-treated cells when compared to control on days 3, 5, and 7 (Figure 2E).

### 2.3. CRISPR-Cas9 Knockout of Gal-3 in MIO-M1 Cells Resulted in Decreased Gal-3 Expression and Release

**Figure 3:**
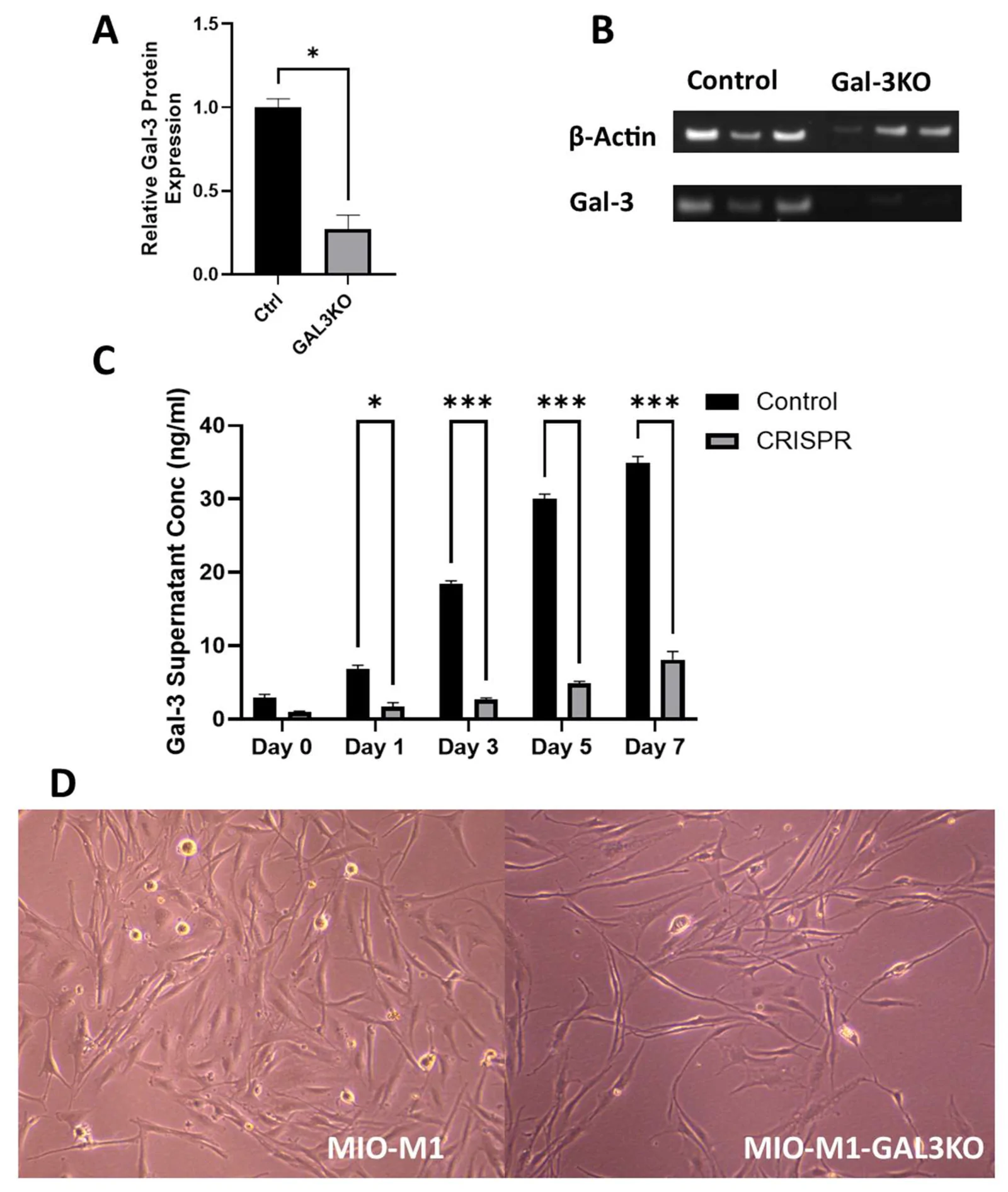
Gal-3 knockout cells generated from the MIO-M1 cell line. (**A**) MIO-M1-GAL3KO cells showed decreased cell lysate Gal-3 concentrations when compared to control MIO-M1 cells as measured by Western blot. (**B**) Representative Western blots. (**C**) MIO-M1-GAL3KO cells showed significantly reduced Gal-3 protein concentration within the supernatant measured using ELISA when compared to control MIO-M1 cells at days 1, 3, 5, and 7 following passaging of cells. (**D**) MIO-M1-GAL3KO cells exhibit thinner and more elongated morphology as compared to control MIO-M1 cells, which tend to be flatter and stellate in shape. *—*p* < 0.05, ***—*p* < 0.001.

Next, a line of Gal-3 knockout cells was generated from MIO-M1 cells using a CRISPR-Cas9 system according to the manufacturer’s instructions. The resultant MIO-M1-Gal3KO cells showed significantly reduced Gal-3 expression within cell lysates when compared to controls (*p* < 0.05, Figure 3A,B). Gal-3 protein concentration within the supernatant of MIO-M1-Gal3KO cells was also reduced when compared to control MIO-M1 cells as measured by ELISA at days 1, 3, 5, and 7 following passaging (*p* < 0.001, Figure 3C). The MIO-M1-Gal3KO cells demonstrated thinner and elongated morphology when compared to control MIO-M1 cells, which exhibited a more typical flat, stellate glial cell appearance in vitro (Figure 3D).

### 2.4. Gal-3 Knockout Cells Show Decreased Proliferation and Migration

**Figure 4:**
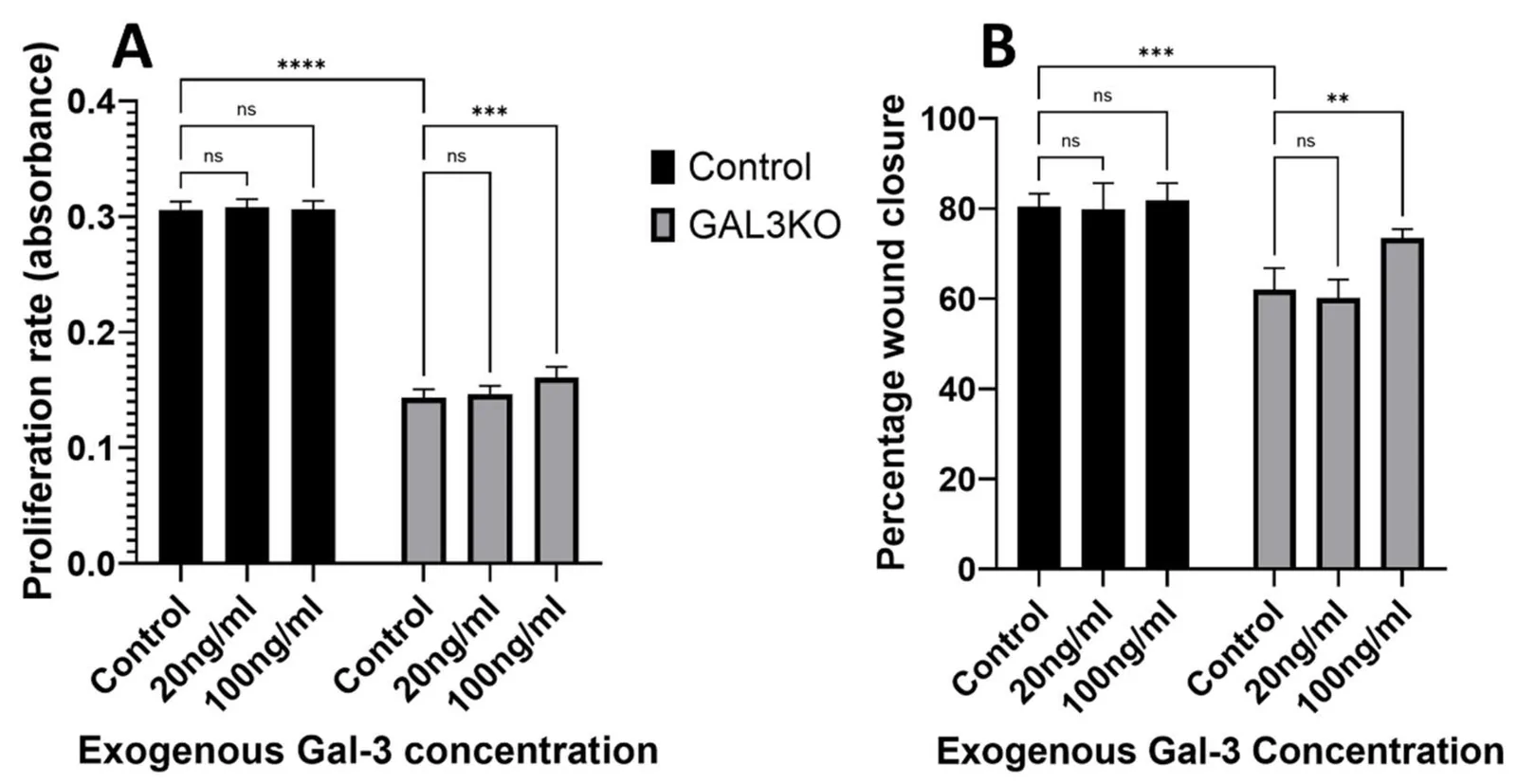
Effects of exogenous Gal-3 on cellular proliferation and migration in MIO-M1 and MIO-M1-GAL3KO cells. (**A**) MIO-M1-GAL3KO cells demonstrated reduced rates of proliferation when compared to control MIO-M1 cells at 7 days. Addition of exogenous recombinant Gal-3 had no effect on control MIO-M1 cells but resulted in a small but significant increase in the rate of proliferation in MIO-M1-GAL3KO cells at 100 ng/mL, with no significant effects observed at 20 ng/mL, *n* = 5. (**B**) MIO-M1-GAL3KO cells demonstrated reduced rates of migration when compared to control MIO-M1 cells at 24 h. Addition of exogenous recombinant Gal-3 had no effect on control MIO-M1 cells but resulted in a small but significant increase in the rate of migration in MIO-M1-GAL3KO cells at 100 ng/mL, with no significant effects observed at 20 ng/mL, *n* = 5. **—*p* < 0.01, ***—*p* < 0.001, ****—*p* < 0.0001.

Gal-3 knockout reduced rates of both proliferation and migration in MIO-M1 cells. At 7 days following cell passage, MIO-M1-GAL3KO cells showed significantly decreased rates of proliferation (*p* < 0.0001, Figure 4A) as compared to control MIO-M1 cells. Addition of exogenous recombinant Gal-3 had no significant impact on the proliferation of control MIO-M1 cells. However, in MIO-M1-GAL3KO cells, exogenous Gal-3 addition resulted in a small but significant increase in proliferation rate when added at 100 ng/mL (*p* < 0.001), but this effect was not observed at 20 ng/mL.

For cellular migration measured at 24 h, MIO-M1-GAL3KO cells showed a similar decrease when compared to control MIO-M1 cells (*p* < 0.001, Figure 4B). Addition of exogenous recombinant Gal-3 had no significant impact on the migration of control MIO-M1 cells, whereas in MIO-M1-GAL3KO cells, this resulted in a significant increase in migration rate when added at 100 ng/mL (*p* < 0.01), but not at 20 ng/mL.

### 2.5. TGF-β Alters the Expression of Sialyltransferase Enzymes

**Figure 5:**
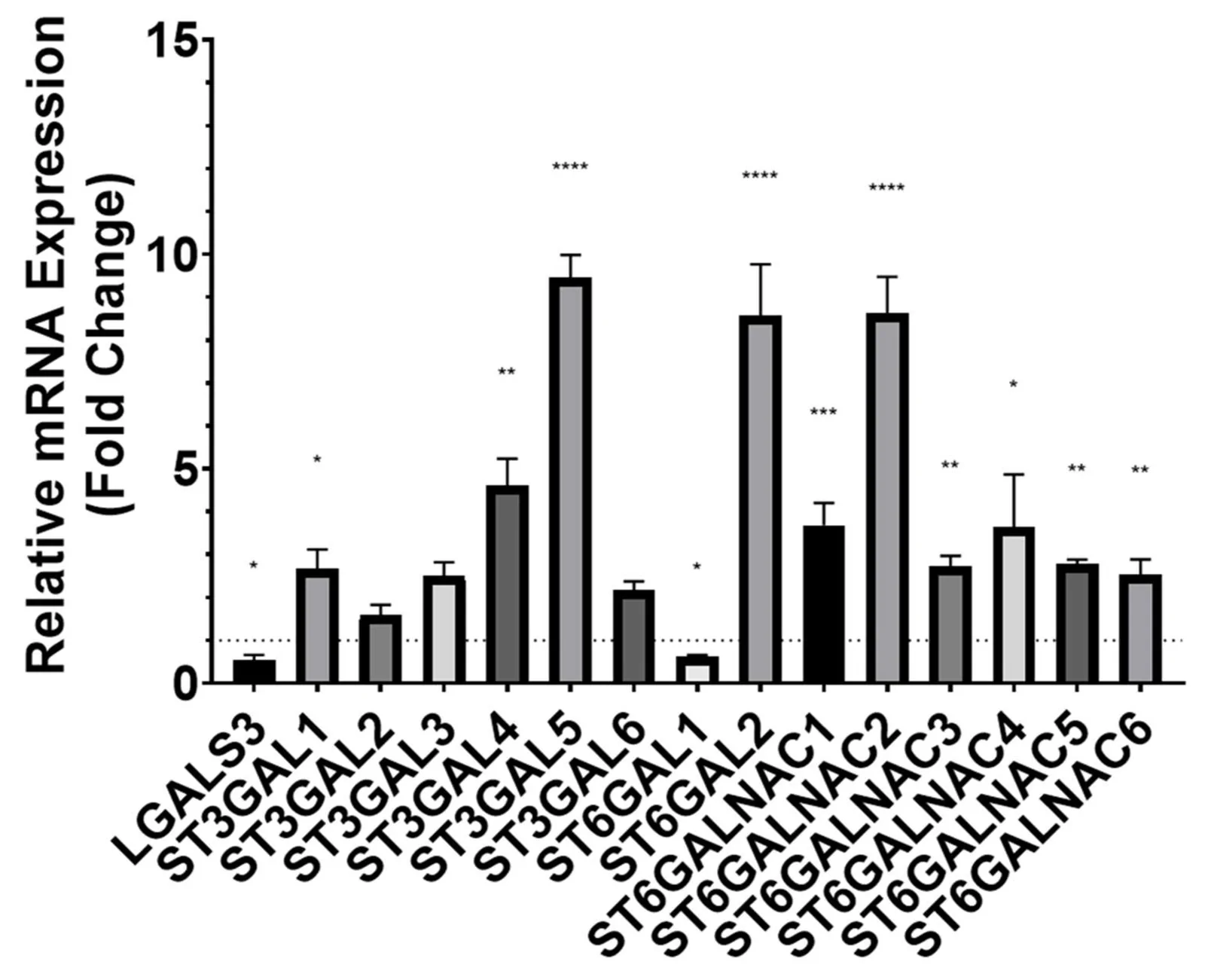
Change in mRNA expression of sialyltransferase enzymes in response to TGF-β treatment of MIO-M1 cells. Within the ST3GAL family, ST3GAL1, ST3GAL4, and ST3GAL5 were significantly upregulated following treatment; within the ST6GAL family, ST6GAL1 was significantly downregulated, whereas ST6GAL2 was significantly upregulated following treatment; all six members of the ST6GALNAC family were significantly upregulated after treatment. *—*p* < 0.05, **—*p* < 0.01, ***—*p* < 0.001, ****—*p* < 0.0001, *n* = 3.

Following the addition of TGF-β_1_ to MIO-M1 cells, mRNA expression of various SiaTs was measured at 24 h. In general, there was an upregulation of STs following TGF-β_1_ treatment, with the notable exception of ST6GAL1 (Figure 5). Amongst members of the ST3GAL family, ST3GAL1 (*p* < 0.05), ST3GAL4 (*p* < 0.01), and ST3GAL5 (*p* < 0.0001) were all significantly upregulated, with ST3GAL5 reaching an over 9-fold increase. The two members of the ST6GAL family showed opposing trends, as ST6GAL1 was significantly downregulated (*p* < 0.05), whilst ST6GAL2 was significantly upregulated (*p* < 0.0001). All members of the ST6GALNAC family were significantly upregulated, most strikingly ST6GALNAC2, which showed an over 8-fold increase (*p* < 0.0001).

### 2.6. Gal-3 Inhibitor TD139 and Neuraminidase 1 Decrease Migration of MIO-M1 Cells

**Figure 6:**
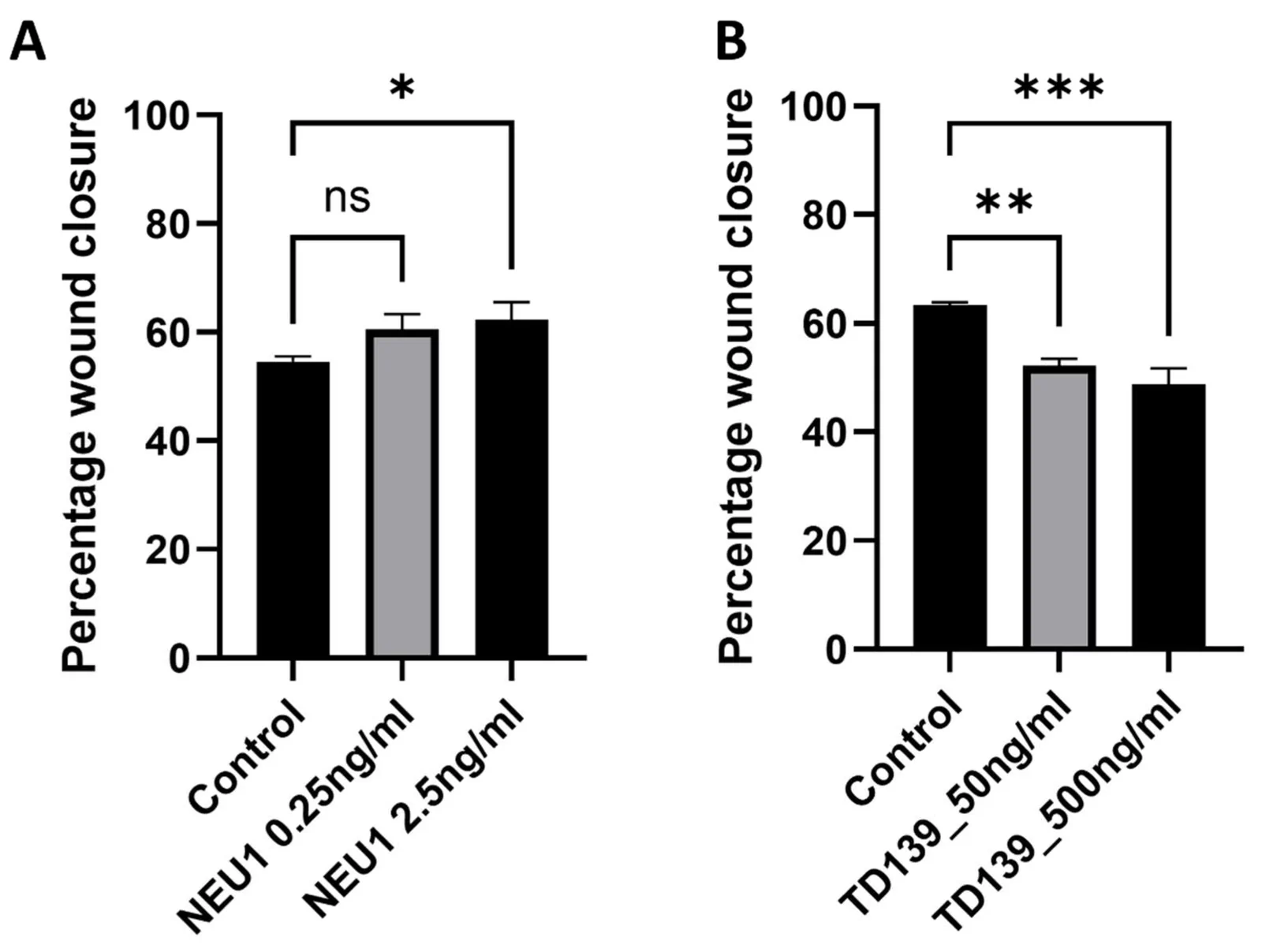
Effects of Neuraminidase 1 (NEU1) and Gal-3 inhibitor TD139 on cellular migration in MIO-M1 cells. (**A**) Treatment with NEU1 at 2.5 ng/mL demonstrated significantly increased rates of migration as compared to control, whilst treatment at the lower concentration of 0.25 ng/mL did not show any significant effect. (**B**) Treatment with the Gal-3 inhibitor TD139 resulted in significantly reduced rates of migration as compared to control at both 50 ng/mL and 500 ng/mL concentrations. *—*p* < 0.05, **—*p* < 0.01, ***—*p* < 0.001.

Recombinant Neuraminidase 1 (NEU1) was added to MIO-M1 cells, at 24 h. Cells treated with NEU1 at 2.5 ng/mL demonstrated significantly increased rates of migration as compared to control (*p* < 0.05, Figure 6A), whilst treatment at the lower concentration of 0.25 ng/mL did not show any significant effect. Treatment with the Gal-3 inhibitor TD139 resulted in significantly reduced rates of migration as compared to control at both 50 ng/mL (*p* < 0.01) and 500 ng/mL (*p* < 0.001) concentrations (Figure 6B).

## 3. Discussion

In this study, downregulation of Gal-3 via CRISPR-Cas9 knockout and the use of the inhibitor TD139 both resulted in reduced rates of migration of MGCs. TD139 has previously been shown to significantly inhibit Gal-3 activity in the context of fibrotic diseases and is currently being investigated as part of a clinical trial for pulmonary fibrosis [23]. The anti-migratory effect of TD139 was also partially rescued via the addition of exogenous recombinant Gal-3 at 100 ng/mL. These findings indicate that elevated Gal-3 concentrations actively promote Müller glial cell migration, which has implications in pathological states such as reactive gliosis, where glial migration is a prominent feature. The mechanisms of Gal-3-mediated cellular migration are likely similar to those of other organ systems where the existing literature has already established Gal-3 as a central regulator of tissue fibrosis [8]. The effects of Gal-3 may be mediated by interactions between Gal-3 and α5β1 integrin to promotes cell-matrix adhesion and modulate wound healing [24,25]. It is interesting to note a change in morphology of MIO-M1 cells after undergoing CRISPR-Cas9 knockout of Gal-3, transitioning from a typically flat, stellate appearance into a more elongated phenotype, demonstrating fewer cytoplasmic processes. This structural remodelling may reflect a change in cell-substrate adhesion given the known function of Gal-3 in stabilising β1 integrin lattices. Alternatively, this finding may also signify a change in cell differentiation state or a cellular stress response. Elucidating the molecular drivers of this phenotypic change would be a compelling subject for future investigation. Previous studies have shown that MIO-M1 cells exhibit expression profiles similar to those of MGCs extracted from induced pluripotent stem cell-derived organoids [26,27]. However, as significant changes can occur following prolonged in vitro cell culture, in vivo confirmation of the findings from the present study would be an important next step.

TGF-β has long been established to be the “master regulator” of fibrosis and migration [28]. This role extends to ocular tissues, where TGF-β has been implicated in a number of ocular diseases. The various functions of TGF-β operate in both beneficial and detrimental processes, highlighting the complex nature of its signalling [29]. In some cases, such as in MGCs during retinal regeneration in zebrafish, the role of TGF-β appears to be biphasic [30]. Interestingly, in this study, we demonstrated that TGF-β reduced the rates of Müller cell proliferation and migration and significantly downregulated Gal-3 expression in MGCs.

TGF-β treatment resulted in lower concentrations of Gal-3 protein within both the cytoplasm and the culture supernatant. This is in keeping with a similar study in nucleus pulposus cells, where TGF-β also downregulated Gal-3 expression via SMAD-mediated decrease in LGALS3 promoter activity [31]. Conversely, a study of human lung fibroblasts showed that Gal-3 induced TGF-β activation via binding to αv integrins [32]. Given that both TGF-β and TGF-β receptors are subject to glycosylation, their interaction may be affected by the degree of sialylation via sialyltransferase (SiaT) expression and subsequent binding with Gal-3. Overall, these findings raise the possibility that TGF-β and Gal-3 may form a complex reciprocal feedback loop involving protein–glycan interaction.

It is worth noting that the decrease in the rate of cell proliferation did not significantly contribute to the decrease in the rate of cell migration, as TGF-β treatment resulted in a 21.3% decrease in the rate of migration over 24 h, whereas the decrease in proliferation was much slower, at 12.6% over 7 days, which equates to around 1.7% per day. An anti-metabolite, such as mitomycin C (MMC), was not used in this study to inhibit proliferation, given the relatively small difference in the rate of migration between groups, and to avoid introduction of potential anti-fibrotic effects of MMC [33].

TGF-β is a central cytokine during gliosis; the results here suggest that constitutive MGC expression of Gal-3 is reduced in the presence of TGF-β. Therefore, it is important to consider other potential sources of Gal-3 protein within the neuroretina during gliotic states, such as activated microglia and infiltrating systemic immune cells [12]. Furthermore, the role of TGF-β in MGC migration and fibrosis may be significantly influenced by the degree of inflammation, which would drive microglial activation and/or influx of circulating immune cells to cause Gal-3 accumulation.

We found TGF-β to be a potent inducer of the SiaT family of enzymes in MIO-M1 cells. ST3- and ST6- families of SiaTs attach α2,3- and α2,6-glycosidic linkages, respectively, and the degree of α2,3- and α2,6-sialylation can in turn influence Gal-3 binding. Following TGF-β treatment, there was a general upregulation of the members of the ST3GAL, ST6GAL and ST6GALNAC families, with the notable exception of ST6GAL1, which was significantly downregulated. The overall pattern following TGF-β treatment is therefore one of hypersialylation, a phenomenon often observed in malignant cells that is thought to be pro-metastatic and causes local immunosuppression [34].

ST3GAL1 has previously been shown to play a role in regulating TGF-β activity via the inactivation of vasorin, a TGF-β binding protein [35]. In addition, ST6GAL1 was found to promote TGF-β-induced epithelial–mesenchymal transition in GE11 cells, whilst ST3GAL4 did not exhibit any such effect [36]. In this study, the reduction in ST6GAL1 expression was accompanied by an increase of over 8-fold in ST6GAL2 mRNA expression. This shift from ST6GAL1 to ST6GAL2 expression following TGF-β treatment is an intriguing finding; whilst ST6GAL1 has been shown to be a major player in cancer that promotes growth and metastasis [37], the mechanistic differences between ST6GAL1 and ST6GAL2 remain poorly understood. ST6GALNAC1 and ST6GALNAC2 were also both upregulated following TGF-β treatment, and similarly to ST6GAL enzymes, ST6GALNAC1 has also been shown to inhibit Gal-3 interaction [38]. There is significant overlap between the function of members of the different SiaT families. Future experiments to determine whether distinct roles of individual enzymes may affect disease processes differently are crucial in elucidating the underlying mechanism of TGF-β-related cellular behaviour.

To better understand the role of sialylation in Müller cell migration, we showed that the addition of Neuraminidase 1 (NEU1) increased the rate of cell migration. NEU1 hydrolyses α2,3- and α2,6-glycosidic linkages, thereby nullifying the effect of sialyltransferases. This finding suggests that a lack of tissue sialylation leads to increased migration of Müller cells, which corroborates a previous study where desialylation of synovial fibroblast cells caused increased cellular migration [39]. Similarly, a recent study demonstrated cell migration to be increased by EGF and dependent on de-sialylation via NEU enzymes, which in turn triggers endocytosis through Gal-3/integrin interactions [40]. Overall, it appears that exposure of MGCs to TGF-β signals a reduction in Gal-3 related activities via Gal-3 downregulation and simultaneous hypersialylation. In contrast, the introduction of neuraminidase appears to counteract the hypersialylation effects of TGF-β.

The findings of this study may be particularly relevant to the pathophysiology of retinal diseases characterised by glial migration and proliferation, such as proliferative vitreoretinopathy (PVR). In PVR, RPE cells and a host of pro-inflammatory mediators disperse from the subretinal space into the vitreous, leading to uncontrolled proliferation, migration and contraction of both Müller cells and RPE cells undergoing epithelial–mesenchymal transition [41]. Given that RPE cells are potent producers of Gal-3 in the normal retina, their displacement into the vitreous likely causes an accumulation of Gal-3, which may in turn drive Müller cell migration as shown in the present study. Translating these findings to human pathology through the detailed analysis of tissue samples from patients with PVR would provide valuable insight into the role of Gal-3 in this disease.

In summary, this study identified Gal-3 as a pro-migratory driver in MGCs during reactive gliosis in a process that is downregulated by TGF-β. As aberrant MGC migration is a key feature in a number of proliferative retinal diseases; targeting Gal-3 or modulating tissue sialylation profiles may offer viable therapeutic strategies for the treatment of these conditions.

## 4. Materials and Methods

### 4.1. Cell Culture

MIO-M1 cells are MGCs derived from cadaveric human retinal tissue which express MGC markers and become spontaneously immortalised. Cells were cultured in Dulbecco’s Modified Eagle Medium (DMEM, Cat No. 31966-047; Gibco, Life Technologies, Carlsbad, CA, USA) containing high glucose, GlutaMAX™, and pyruvate. This was supplemented with penicillin at 20 U/mL and streptomycin at 20 µg/mL (Cat No. 15070-063; Gibco, Life Technologies), as well as 10% Foetal Calf Serum (FCS; Biosera, Boussens, France). The cells were incubated at 37 °C in a 5% CO_2_ atmosphere. Cell passaging was carried out using TrypLE™ Express Enzyme (Cat No. 12604-013; Gibco, Life Technologies), followed by washing the cells with phosphate buffer solution (PBS) and centrifuging at 1500 RPM to obtain a cell pellet [42].

Additional reagents used during cell culture included TNF-α (Cat No. 300-01A-50UG, ThermoFisher, Waltham, MA, USA), IL-1 (Cat No. 200-01B-10UG, ThermoFisher), IL-6 (Cat No. 1019, Proteintech, Rosemont, IL, USA), TGF-β1 (Cat No. 100-21-10UG, ThermoFisher), TGF-β2 (Cat No. 100-35B-10UG, ThermoFisher), IFN-γ (Cat No. 1301, Proteintech), Gal-3 protein (Cat No. 450-38-50UG, ThermoFisher), Neuraminidase 1 (Cat No. ab131685, Abcam, Cambridge, UK) and TD139 (Cat No. ab286972, Abcam).

### 4.2. Cell Proliferation Assay

Cell proliferation was assessed using the MTT (3-(4,5-dimethylthiazol-2-yl)-2,5-diphenyltetrazolium bromide) assay kit (Cat no. ab211091, Abcam) according to the manufacturer’s instructions. Briefly, 4000 cells/well were seeded onto a 96-well microplate at the start of each assay. These were allowed to adhere overnight, and treatment with cytokines or exogenous Gal-3 was carried out on the following day. Absorbance was recorded at day 7 using a microplate reader at 590 nm wavelength.

### 4.3. Cell Scratch Migration Assay

Cell migration was assessed with the scratch assay, using the AutoScratch Wound Tool (Agilent Technologies, Santa Clara, CA, USA) [43]. Briefly, 10,000 cells were seeded per well onto a 96-well microplate (Costar 3598, Corning, NY, USA) and allowed to reach 80–90% confluency, which usually required 2–3 days. The AutoScratch Wound Tool was then used to ensure that a consistent wound was created at the centre of each well. The wells were then washed with PBS before conditioning media were added. Imaging of the wound was carried out using Cytation 8 (Agilent Technologies, USA) immediately following the initial scratch and again at 24 h. The area without cells was measured using ImageJ software (version 1.54g, Java, Austin, TX, USA). Anti-metabolites such as mitomycin C were not used during the scratch migration assay.

### 4.4. CRISPR-Cas9 Knockout Cell-Line Generation

CRISPR-Cas9 pre-cloned guide RNA cassettes targeting human LGALS3 were purchased from Origene (Cat no. KN208785RB), and knockout of MIO-M1 cells was performed according to the manufacturer’s instructions. Briefly, 4000 MIO-M1 cells were seeded per well onto 24-well plates and allowed to reach 50% confluency. At this point, 1 µg guide RNA and 1 µg donor DNA were added to the standard culture media, followed by 6 µL TurboFectin 8.0 (Cat no. TF81001, Origine Technologies, Rockville, MD, USA). The cells were passaged at 90–100% confluency at a 1 in 5 ratio over the following 3 passages. Puromycin (Cat no. P8833, Merck, Rahway, NJ, USA) was then added at 750 ng/mL, as this was previously determined to cause 100% cell death in control MIO-M1 cells. The surviving cells following puromycin selection were grown until confluent and passaged as per control MIO-M1 cells outlined above.

### 4.5. ELISA Assay

A total of 4000 MIO-M1 cells were seeded per well onto a 96-well microplate (Costar 3598, Corning, NY, USA) in standard culturing media (Section 4.1). At days 1, 3, 5 and 7, 200 µL of supernatant was collected from each well and stored at −20 °C. Supernatant collection for day 0 was collected at 4 h following cell seeding to allow attachment. Gal-3 concentrations of cell culture supernatant samples were measured using Human Galectin-3 Quantikine ELISA Kit (Cat no. DGAL30, R&D Systems, Minneapolis, MN, USA) according to the manufacturer’s instructions. Briefly, 50 µL of each sample was added to a well of the ELISA test plate and allowed to incubate for 2 h at room temperature. A 50 µL aliquot of Gal-3 serial dilution standard was also added at the same time, and all samples were repeated in triplicate. The contents of each well were then aspirated and washed 4 times using 400 µL of the provided wash buffer. Then, 200 µL of Gal-3 Conjugate was added to each well and incubated for an additional 2 h at room temperature. The wells were then washed a further 4 times using 400 µL of the provided wash buffer. Next, 200 µL of Substrate Solution was added to each well and incubated for 30 min at room temperature whilst protected from sunlight. Following this, 50 µL of Stop Solution was added to each sample, and the absorbance of the ELISA plate wells was measured immediately at 450 nm using a spectrophotometer (Spark Multimode Reader, Tecan, Männedorf, Switzerland). The concentration of each sample well was then calculated against the standard curve generated by serial dilution samples using log/log curve-fit.

### 4.6. RNA Isolation, Reverse Transcription and Quantitative PCR

The isolation of RNA was performed using the RNeasy Plus Mini Kit (Cat No. 74134; Qiagen, Hilden, Germany) as per manufacturer’s instructions. Final RNA elution was carried out using 30 µL of nuclease-free water, and the final RNA concentration was measured by spectrophotometry (Nanodrop-1000, Thermo Fisher Scientific, Pittsburgh, PA, USA).

Reverse transcription (RT) was carried out using the SuperScript^®^ IV First-Strand Synthesis System (Cat No. 18091050, Thermo Fisher Scientific). A 200 µL reaction tube contained 500 ng or 1 µg RNA, 1 µL 50µM Oligo d(T)18 primer (Cat No. 18418-012; Life Technologies), 1 µL 10 mM dNTP mix (Cat No. U151A; Promega, Madison, WI, USA), and nuclease-free water to reach a final volume of 13.5 µL. This initial reaction mix was briefly vortexed and centrifuged, then heated to 65 °C for 5 min using a thermal cycler (Mastercycler^®^ Pro: vapo protect; Eppendorf, Stevenage, UK) to allow for annealing to occur, followed by at least 1 min at 4 °C. Next, 4 µL 5× SSIV buffer, 1 µL DTT buffer, 0.5 µL RNasin^®^ Plus RNase inhibitor (Cat No. N2611; Promega, Madison, WI, USA) and 1 µL SuperScript^®^ IV reverse transcriptase enzyme were added to the initial reaction mixture. This mixture was again briefly vortexed and centrifuged, followed by incubations at 55 °C for 10 min and 80 °C for 10 min.

Quantitative polymerase chain reactions (qPCR) were carried out using SYBR^®^ Green JumpStart™ Taq ReadyMix™ (Cat no. S4438, Sigma-Aldrich, Dorset, UK), according to the manufacturer’s instructions. This consisted of 10 μL SYBR green reagent, 0.25 μL indicator dye, 1 μL each forward and reverse primers (Table 1) and 10 ng cDNA made up to 9.75 μL using nuclease-free water. Reactions were carried out on MicroAmp^®^ Optical 96-Well Reaction Plates (Cat no. N8010560, Thermo Fisher), and each condition was repeated in triplicate. A QuantStudio™ 6 Flex Real-Time PCR System (Applied Biosystems, Waltham, MA, USA) was used as the thermo cycler. Each 96-well plate was sealed with an adhesive film to avoid evaporation and centrifuged prior to placing in the thermo cycler machine.

Thermo cycling was programmed to initially hold at 50 °C for 2 min and then 95 °C for 10 min, followed by the PCR step for 40 cycles at 95 °C for 15 s and 60 °C for 1 min, with a final melt curve step at 95 °C for 15 s, 60 °C for 1 min and a dissociation stage at 95 °C for 15 s. Data were collected between the 60 °C and 72 °C stages of each cycle.

**Table 1:** Quantitative PCR Primers.

Target	Forward Primer	Reverse Primer
GAPDH	GTCTCCTCTGACTTCAACAGCG	ACCACCCTGTGCTGTAGCCAA
LGALS3	TGATTGTGCCTTATAACCTG	ATGACTCTCCTGTTGTTCTC
ST3GAL1	GAAACTCCAGCGTGTCTCCA	CATAGGCTGAGTGACCGTCC
ST3GAL2	AGAAGCTGTTCCAGATAGTG	ATCCTCATGATGAAGTTGTG
ST3GAL3	CAGTGGTTCTTTCCTTTGAC	TAATTCAGCAGGCAGTTTAG
ST3GAL4	CACGGAAGATTAAGCAGAAG	CATAGTAGTGAATGGTCTGC
ST3GAL5	AGTTCTCCAGTAAAGTCCAG	CCTTATCACAACATCGAACTG
ST3GAL6	CAGAAACCTAAACACCCAAC	CATTCCCATAGTAGTGCAAAG
ST6GAL1	CTACCACCCGCTGCTCTATG	GTGTGGCTTTTCCAAGCAGG
ST6GAL2	ACCCAAATCAGCCATTTTAC	TCCAATGAAACCAGAAGATG
ST6GALNAC1	TCCGATACATGAAGAACAGG	CATAGTAGTGATCAGAAAAGCG
ST6GALNAC2	CCCATGACTATGTATTCAGAC	CACAGTGAAACCATAGAAGG
ST6GALNAC3	AAGAGAAAGTCTGTGATTGC	TATCCATAGTGAGTTCGAAGG
ST6GALNAC4	CTACTGCAGGGAGAAGAG	TTCTCAGTGATGAAGCGG
ST6GALNAC5	CAGGAAGATATCCAACACTTG	TGCCATAAACATTGATCCTG
ST6GALNAC6	AATGAGTAGCAACAAAGAGC	GTAATGGAAGACCTCATTGG

### 4.7. Protein Isolation and Western Blotting

Protein extraction was performed on cell pellets by adding 100 µL radio immunoprecipitation assay (RIPA) lysis buffer (Cat No. R0278; Sigma Aldrich, St. Louis, MO, USA) containing 3 mM sodium orthovanadate, 1 mM phenylmethane sulphonyl fluoride (PMSF), 0.5 mM dithiothreitol (DTT) and 10 µL protease inhibitor cocktail (Cat No. P8340; Sigma Aldrich). Protein gel electrophoresis was carried out using 15-well 4–12% Bis-Tris polyacrylamide gels (Cat No. NP0336; Thermo Fisher Scientific) and MES SDS running buffer (Cat No. NP0002; Thermo Fisher Scientific). For each sample, 10 µg of protein made up to 9.75 µL was added to 3.75 µL of 4× LDS sample loading buffer (Cat No. NP0007; Thermo Fisher Scientific) and 1.5 µL of 10× reducing agent (Cat No. NP0009; Thermo Fisher Scientific). The loading protein mixture was briefly vortexed, centrifuged, then denatured at 80 °C for 10 min into primary structures.

Protein transfers were performed using 0.45 µm polyvinylidene fluoride (PVDF) membranes (Cat No. IPFL00010; Merck Millipore, Darmstadt, Germany) using the Trans-Blot^®^ SD Semi-Dry Transfer Cell (Bio-Rad Laboratories, Hercules, CA, USA). The PVDF membranes containing the transferred proteins were placed in 10 mL of EveryBlot Blocking Buffer solution for 1 h (Cat No. 12010020; Bio-Rad, USA). The membrane was then incubated in further blocking solution containing the primary antibody for the protein of interest overnight at 4 °C on a shaker. On the next day, the membrane was washed 3 times using 0.1% Tween-20 diluted in TBS for 30 min. For chemiluminescence detection, the membrane was incubated in blocking solution containing 1:5000 of the corresponding secondary species-specific antibody conjugated to horseradish peroxidase (Jackson ImmunoResearch Laboratories Inc., West Grove, PA, USA) for 1 h at room temperature. Chemiluminescent substrate (Cat No. WBLUR0500; Millipore Corporation, Billerica, MA, USA) was added for 2 min, and the final membrane was imaged using the Bio-Rad ChemiDoc Imager (Bio-Rad, USA). For fluorescence detection, the membrane was incubated in blocking solution containing 1:5000 of the corresponding secondary species-specific fluorescent antibody and washed 3 times further for 30 min, and the final membrane was imaged using the Bio-Rad ChemiDoc Imager. Antibodies used in this experiment included Vimentin (Rabbit, ab92547, Abcam) and Gal-3 (Rabbit, ab76245, Abcam).

### 4.8. Immunocytochemical Staining

Thirteen-millimetre microscope slide coverslips were placed in 24-well plates and pre-coated with 250 µL Matrigel (Cat No. E6909; Sigma Aldrich) for 2 h. At the end of each experimental period, the media were aspirated from the wells, and 4% paraformaldehyde (PFA) was used to fix the cells for 5 min. The wells were washed 3 times with PBS and covered in blocking solution containing Tris-buffered saline (TBS), 0.3% Triton and 5% donkey serum at room temperature for 1 h. This blocking solution was then replaced by a primary antibody containing blocking solution and incubated overnight at 4 °C. On the following day, the wells were washed 3 times for 5 min with PBS, then incubated in donkey-derived secondary antibody labelled with Alexa-Fluor fluorochromes (Jackson ImmunoResearch Laboratories, PA, USA) diluted in blocking solution for 3 h at room temperature. The cells were washed a further 3 times and stained with 4’, 6-diamidino-2-phenylidole (DAPI; Cat No. D9542; Sigma Aldrich) diluted at 1:5000 in PBS for 2 min, then washed with PBS and MiliQ water for 1 min each. Finally, the coverslips were removed from the 24-well plates and mounted to microscope slides using Vectashield (Vector Laboratories, Newark, CA, USA) and sealed with nail varnish.

## Data Availability

The raw data supporting the conclusions of this article will be made available by the authors on request.
